# Balanitis

**Published:** 1875-02-01

**Authors:** F. K. Bailey

**Affiliations:** Knoxville, Tenn.


### MEDICAL EXAMINER.

A

*Semi-Monthly Journal of Medical Sciences.*

EDITED BY N. S. DAVIS, M.D., AND F. H. DAVIS, M.D.

No. III. CHICAGO, FEB. 1, 1875. VOL. XVI.

### Original Communications.

### BALANITIS.

BY F. K. BAILEY, M. D., KNOXVILLE, TENN.

FREQUENTLY, upon a person calling for medical or surgical advice, we will at once diagnose the case from appearances which are obvious at the time.

What may be developed subsequently, it is often impossible to determine. The following case was diagnosed as balanitis, and at first it appeared susceptible of immediate cure.

April 8th, 1873—I. B. ——, aged thirty-five, of sanguine bilious temperament, well formed and muscular, occupation, stone-cutter, called upon me at the office. States that he has had sores upon the membrum virile for a week or more, and fears they may be chancrous. Found on inspection, that at the base of the glans, and bordering upon the preputial reflection, there was a row of small ulcerated spots associated with papules still unbroken, extending from frænum to frænum.

Considerable discharge of rather offensive character, and copious; cellular tissue slightly infiltrated in the prepuce; very sensitive and painful; no inguinal tenderness. States that he had chancres and bubo in one groin within a year, but had supposed himself cured.

Had connection about fifteen days previously with a woman who was not above suspicion, and hence his fears.

Besides the local troubles, I found his tongue coated, the skin and conjunctiva yellow; pulse about ninety; bowels confined; urine red and hot.

Pres. comp. cath. pills, and a weak solution of carbolic acid as a wash. I also touched the affected parts with carbolic acid and water equal parts.

12th.—Has called in two or three times, when I found the local lesion unchanged, except increased œdema of the prepuce, and soreness unabated; still yellow, and tongue coated. Added acetate lead to the carbolic wash, and gave calomel and Dover's powder, grs. iij. of the former, and grs. v. of the latter, to be taken night and morning.

21st.—Visited him at his residence, and found the prepuce much swollen and painful. Glans not swollen, and liquids can be made to reach the affected parts. No active inflammation, but parts very sensitive to the touch; bowels open, but skin hot; pulse ninety or more; could not sleep for two nights. Left pulv. Doveri, to be taken every four hours, and continue wash.

24th.—Visited patient, and found that on yesterday, hæmorrhage had occurred, which soon stopped; also, that at 3 a. m. to-day, the bleeding returned, and continued for three hours; reports the loss of a large quantity of blood from the penis, and some from the nose. Saw him at 9.30 a. m., and found the pulse slow, (less than seventy,) soft and small; countenance clear; tongue cleaning; complains of soreness of the gums, which may be a result of mercurial action; had fever every day till to-day; I had sent him quinine on the 22d and 23d, which produced ringing in the ears, and last night he says he was considered delirious by the family. Find the swelling of prepuce much diminished, and on the right side of the glans anteriorly, a small abraded spot, from which the blood had flowed.

There is a pinched and rather bluish appearance to the glans, urine clear, and more abundant; bowels open from castor oil taken last night; free from pain, and has some appetite; left powder of gallic acid to be taken every three hours, alternated with small doses of quinine; also left Monsel's solution to apply, should bleeding return; diet to be simple, but nutritious.

25th.—Very much improved; can sit up, and the affected parts appear to be healing; no return of the hæmorrhage; to take one gr. quinine three times daily; directed him to touch the ulcerated surface with nit. silver stick once a day.

28th.—Patient called at my office, having walked one and one-half miles; found him much improved in general appearance; tongue cleaning; no fever, and free from pain; on examining the membrum virile, found deep ulcerations upon the glans, which appear to have extended the whole circumference; pus healthy, and the prepuce less swollen, and free from inflammation.

Dressed the parts by means of a small rope of cotton pushed under the prepuce to the ulcerated surface, and saturated with a solution of carbolic acid; gave six blue pills, to be taken night and morning till the bowels move freely. I should have observed that the glands in each groin were somewhat tender, and slightly enlarged. Also prescribed as follows:

R.—Iod. Potassii, ʒ ij.

Liquor Iod. Ferri, ʒ iij.

Syr. Zinzibius,

Syr. Sarsæ Compos, a. a. } ℥ j.

M.—Sig. Teaspoonful at each meal and at bed time.

30th.—Bowels open, but says the stools were very lumpy; ulcerations much as on the 28th. Pus healthy. Preputial œdema lessening; can expose the morbid surface much better than at any previous time. Touched the ulcerated surface with nit. silver, and dressed with cotton wet with sol. carbolic acid. To take castor oil to-night.

May 2d.—Called; much the same; ulcerations not extending; a chancrous looking sore on the front of glans, and another on the frænum.

Applied nit. silver, and dressed as before; tongue cleaner; no appetite, and complains of debility; bowels open. Pres. as follows.

| | | |
|---|---|---|
| R.—Aromat. Sul. Acid, | | ʒ ij. |
| | Sulph. Cinchoniæ | gr.i |
| | Tr. Cinch. Compos. | ℥ iiss. |

M. Sig. Teaspoonful before each meal.

3d.—Appetite improved; ulcerations not extending, but indisposed to heal. At the suggestion of a medical friend applied weak dilutions of nit. acid, and gave pills as follows:

| | | |
|---|---|---|
| ℞ | Sulph. Quinine, | grs. xvj. |
| | Pulv. Opii. | " iv. |
| | Calomel. | " viij. |

M.—F. Pil. No. 8. Sig. One every four hours.

12th.—Attempted to work a few days, but found that muscular exertion caused some excitement in the parts. But little change in the ulcerated surfaces; applied stick nit. silver, and continued the carbolic dressings.

16th.—General appearance more favorable to healing, but there is still a tardiness not desirable; small ulcerative spots near the frænum, and upon the glans. The integumentary tissue of the prepuce less œdematous; some appetite, and bowels not constipated; but cannot sleep well; applied dry calomel, and gave ten grs. as a cathartic, followed with castor oil.

18th.—Much the same; insomnia, mostly from soreness of the parts; gave brom. potassi. in doses of 15 grs. at night.

22d.—Still indisposed to heal; has taken quinine for a few days past, with some improvement in appetite; has been at work since the 19th; applied iodoform to the ulcerations, and dress with sol. carbolic acid as before; cont. quinine and brom. potass.

23d.—Slight improvement in appearance of ulcers; cont. iodoform and quinine.

24th—Induration of the integuments increased, with soreness, and the ulcers still indolent; applied opium in powder, with sub. nit. bismuth.

26th.—Slept better for two nights past, there being but little pain; induration somewhat lessened; cont. opium dressing.

27th.—Induration extending upwards on the penis, upon the left dorsal aspect; ulcers about the same; applied calomel with opium.

29th.—Was sick yesterday from headache, chills, etc.

Called this a. m., when I found the ulcers unchanged, still painful at night. Prescribed black-wash, and as a temporary dressing used opium and salicin. Internally the following:

| | | |
|---|---|---|
| ℞ | Nitro-Muriatic Acid, | ʒ ij. |
| | Sul. Quinine, | gr.i. |
| | Tr. Cinch. Compos., | ℥ iiss. |

M.—Sig. Teaspoonful morning and evening.

To apply to-night, also opium and salicin.

30th.—Much the same; the ulceration in the cellular tissue of the prepuce disposed to continue, appears to be working through to the surface from the original point of commencement. Applied sol. permanganate potash to the ulcers, all of which are indisposed to heal. To continue acid mixture.

31st.—Morbid surfaces cleaner and more natural in color, except the sinuous canal into the cellular tissue upon the left anterior of the penis. Appearances as if the integument which for some time has been indurated, may all slough off. Poured in solution permanganate potash, which caused more smarting than on yesterday. Patient having had chills for three nights past, followed with fever and sweating; p. m., quinine in four gr. doses till twelve grs. are taken, and to continue acid mixture. Reports that there are two discharges from the bowels every twenty-four hours, thin and bilious; appetite still indifferent.

June 2d.—Called at my office; had fever last night, and the pulse is one hundred; tongue still slightly coated; no change in ulcers; near the frænum on each side, the ulceration threatens to invade the urethral walls; bowels are loose, result of the acid mixture which he is to suspend, and take

| | | |
|---|---|---|
| ℞ Sulph. Quinine, | grs. | xxiv. |
| Pulv. Opii, | " | iv. |
| Calomel, | " | x. |

M.—F. Pulv. No. 8.
Sig. One every four hours. Also

| | |
|---|---|
| ℞ Labarraques Sol. | ℥ vj. |
| Permanganate Potassæ, | ʒ iss. |

M.

Use one part to three of water as injection, with directions to keep the whole ulcerated surface free from pus; to keep still and not attempt to come to the city.

11th.—Since the 2d inst., the patient has been visited at his residence by Dr. D. T. Boynton, and until today I had not seen him.

The ulceration has continued to spread, and the integument appears much infiltrated still. Sloughing of the prepuce has continued, and the parts present a rough appearance; has taken quinine in two gr. doses every four hours with opium; has slept more than at one time, and has some appetite. The chlorine solution has been continued, with permanganate of potash, introduced by means of a long speculum syringe.

17th.—Visited patient with Dr. Boynton; since the last report, much of the infiltrated integument has sloughed away, and the parts externally appear more regular in form.

There is still a sinus upon the dorsum, a little left of the mesian line, which threatens to invade the body of the organ.

Dr. B. made an incision in the direction of the sinus, laying the whole open, thereby relieving an apparent strangulation which lay across the dorsum, and near the corona glandis. On the whole there is evident improvement, and I may add that a few days ago hæmorrhage occurred, which was controlled by sol. persulphate of iron.

To continue tonics and opiates, with chlorinated soda.

July 1st.—Found general condition unchanged; free discharge from the openings, and the cellular tissue still infiltrated. Treatment to be continued.

13th.—Did not see the case till today. The body of the penis still much enlarged, and the cellular tissue involved to within an inch of the perineum. General health not improving. There is an ulcer upon the anterior right tibial region at about the lower third, which assumes an unhealthy appearance.

Indisposed to heal; has been taking tr. mur. ferri. with quinine, calomel occasionally when the bowels are torpid, opium P. R. N.

The case being under the care of Dr. Boynton, I did not see the man until

August 9th.—Found the organ enlarged, and a deep sinus opening upon the surface on the right side. Pus appears to come from it more free than at any former period; has been injecting sol. carbolic acid in sweet oil, and taking tonics with brom. potassii. Ulcer upon the leg extending, and is quite deep. Despondent and impatient; to continue treatment, and to dress the leg with stimulating applications.

13th.—During the absence from town of Dr. Boynton, I have visited this case of late, and to-day called Dr. Boyd to accompany me. Decided to lay open the sinus, and therefore introduced a director as far as it would reach, passed a probe pointed bistoury in, and cut out about an inch in extent; there was some hæmorrhage, but it was soon stopped by pressure; dressed so as to keep up apposition of the sinus walls. To continue tonics.

17th.—The opening made by the bistoury is wide and gaping, but the general appearance not any more unfavorable. Simple dressings.

Oct. 1st.—Owing to illness, I had not been able to see this man, and he passed into other hands; I learn however that the penis is healed; no further discharge of pus since the operation; ulcer upon leg extending in size, and not improved in appearance.

Jan., 1874.—I have lately learned that this man went to the Hot Springs, Arkansas, during the autumn, and returned much improved; no return of the ulcerations upon the membrum virile, and the sore on the leg healed.

He was treated by means of large doses of iodide of potassium, which was the principal internal medication. I have not seen the man for four months, but understand his health is fast improving.

The above case is given somewhat in detail, as an instance of persistent ulceration and sloughing in a locality which for obvious reasons is slow to heal when diseased.

The man was broken down in health in consequence of his excesses, having been a subject of syphilis before the war. He was a soldier in the Federal army, and since his discharge therefrom, has been far from regular in his habits. On his first calling upon me, the morbid appearances described were such as we often find, and defined as balanitis.

The sequel proved that a deep-rooted affection had to be dealt with.

The terms balanitis and posthitis are alike applicable, for both the glans and prepuce were involved. An important feature in the case was the persistent obstinacy of the ulcerating tissue. It seemed at one time that the whole organ was bound to slough off; every local application appeared to have no effect, and only constitutional remedies were effective at last.

Ordinary gonorrhœal balanitis is quite common, and unless complicated with phymosis, easily cured. During the last spring, I met with three cases, and all negro boys not over fifteen years old, in which the prepuce was drawn over the glans, and could not be retracted when they called in. One of the number ceased his calls when told that an operation might be necessary. In the others I operated by cutting the prepuce with a bistoury, to relieve pressure. One soon got well, but the other was protracted by an œdematous condition of the whole organ, and a gleety discharge which was very obstinate. There was also induration in the inguinal glands upon the right side, and I had reason to believe there had been chancrous sores previously to his coming under my care. I have met with many cases of excoriations upon the glans in negroes, whose habits are filthy, and that cohabit with a class of women who are never free from some disgusting condition of the genitals, such as herpetic eruptions, condylomata, and eczematous sores around the vulva, as well as vaginal leucorrhœa, and gonorrhœal discharges.

